# Study on physiological changes and response mechanism of *Cerasus humilis* under alkali stress

**DOI:** 10.3389/fpls.2025.1586093

**Published:** 2025-05-21

**Authors:** Meitong Pan, Shan Jiang, Lengleng Ma, Junbai Ma, Chenzhuo Yue, Lingyang Kong, Danli Wang, Wei Ma, Xiubo Liu, Weichao Ren

**Affiliations:** ^1^ College of Pharmacy, Heilongjiang University of Chinese Medicine, Harbin, China; ^2^ Functional Natural Products Research Center, Yichun Branch of Heilongjiang Academy of Forestry, Yichun, China; ^3^ College of Jiamusi, Heilongjiang University of Chinese Medicine, Jiamusi, China

**Keywords:** gene function, gene family, SOD gene, plant antioxidant, abiotic stress

## Abstract

**Introduction:**

*Cerasus humilis* has high economic and nutritional value, but at the molecular level, there are few studies on salt-alkali stress of *C. humilis*, and no one has reported the response mechanism of the oxidation system of *C. humilis* under abiotic stress.

**Methods and results:**

In this research, transcriptomic and metabolomic analysis showed that *C. humilis* had a wide range of metabolic activities under alkali stress, and antioxidant enzymes played an important role in response to alkali stress. *ChWRKY29* and *ChWRKY34*, which are likely to respond to alkali stress, were screened based on transcriptomic data and phylogenetic relationship, and their direct regulation on downstream *ChMSD2* and *ChCSD2* genes were verified by yeast single hybridization experiment. Combined with heat map and qPCR analysis, *ChWRKY29* and *ChWRKY34* may regulate the up-regulation of *ChMSD2* and *ChCSD2* gene expression under alkali treatment, and further affect the antioxidant capacity of plants in response to alkali stress. The analysis of *ChSOD* gene family showed that 9 *ChSODs* were identified from *C. humilis*, which were the closest relatives to *Pyrus bretschneideri*. There are a certain number of cis-acting elements in the *ChSOD* promoter region for hormone and abiotic stress, and there is no tandem replication between *ChSOD* genes, but only one fragment replication. Fragment repetition may be the main driving force for the evolution of *SOD* gene family in *C. humilis*, and the results of interspecific collinearity analysis indicate that *C. humilis* and *Malus pumila* are most closely related.

**Discussion:**

In this study, the mechanism of alkali resistance of *C. humilis* was discussed, which provided reference for exploring the mechanism of alkali resistance of rosaceae, in order to provide scientific theoretical basis for expanding the cultivation range and development and utilization of *C. humilis*.

## 1 Introduction

Soil salinization is the process of accumulation of salt and alkali in soil. Soil salinization is a global environmental problem that threatens agricultural and forestry production. The formation of salinized soil is affected by many natural and human factors such as climate, groundwater, vegetation, unreasonable tillage, and so on. The study shows that the global salinized land area is nearly 954 million hectares. When the degree of soil salinization is high, a high saline-alkali environment will be formed (Liu and Wang, 2021), and the content of alkaline substances in the soil such as sodium carbonate and sodium bicarbonate increases, so that the soil pH value increases. Plants growing in such soil will be subjected to alkali stress, that is, the high pH value of the environment around the plant roots and the excessive alkaline ions have an adverse effect on the plant. The harm of salinization to plants is mainly caused by physiological drought of plants. In alkaline environment, especially in higher pH environment, not only the cell membrane structure is damaged, but also the normal physiological metabolism of plants will be affected. The damage of cell structure and metabolic disorders make the plant gradually damaged, and the accumulation of damage leads to the death of the plant. On the other hand, the growth of plants subjected to alkali stress is inhibited, the biomass is reduced, and the vegetation coverage is decreased, which makes the soil more vulnerable to erosion such as wind erosion and water erosion, accelerates the migration and diffusion of salt and alkali content in the soil, and further aggravates the land salinization. Under alkali stress, plants may also secrete some organic substances through their roots, which promotes the dissolution and migration of alkaline substances in the soil, and aggravates land salinization to a certain extent. Therefore, it is of great significance to study the alkali-tolerant mechanism of plants to cultivate alkali-tolerant plants, and to further improve the utilization rate of land resources and improve the ecological environment (Sun et al., 2016; Wang et al., 2017).

Reactive oxygen species (ROS), an inherent byproduct of aerobic metabolism within plant cells, exhibit a dual function in the context of plant growth, development, and stress response mechanisms (Gupta et al., 2019; Mittler, 2002). In the context of alkaline stress, there is an elevation in reactive oxygen species (ROS) within plants. These ROS can interact with cellular components such as lipids, proteins, and nucleic acids, resulting in lipid peroxidation, protein denaturation, and nucleic acid damage. If not promptly eliminated, these effects can lead to structural and functional damage to cells, potentially culminating in cell death. This scenario poses a significant threat to the plant’s survival (Bose et al., 2013). In biological systems, various types of antioxidant enzymes are present. Among these, eroxide dismutase (SOD), catalase (CAT), peroxidase (POD), and glutathione peroxidase (GPx) stand out for their crucial role in the elimination of free radicals (Zheng et al., 2023). The main role of this process is to unevenly transform superoxide radicals into hydrogen peroxide and oxygen, accomplished by disproportionation (Gill et al., 2015). Among higher plants, *SOD* gene is classified into three types based on their binding metal cofactors: Cu/Zn-SOD, Fe-SOD, and Mn-SOD (Li et al., 2021). Among them, Cu/Zn-SOD mainly exists in mitochondria, chloroplasts and cytoplasm, while Mn-SOD mainly exists in mitochondria and peroxisome. Fe-SOD predominantly occurs in the compartments of mitochondria, chloroplasts, and peroxisomes. Conversely, Nickel Superoxide Dismutase (Ni-SOD) is primarily identified in Streptomsupyces, cyanobacteria, and marine organisms, and its presence in plants has yet to be documented (Dupont et al., 2008; Schmidt et al., 2009).

*Cerasus humilis* is mainly distributed in the northern part of China. *C. humilis* has a long reproductive period, beautiful appearance and high ornamental value. The fruit of its cultivar is edible and rich in calcium and iron, making it a promising third-generation fruit tree. *C. humilis* seed is a famous Chinese medicine – Yu Li Ren. The stems and leaves are rich in calcium and are good fodder for cattle and sheep. *C. humilis* is particularly tolerant to drought, cold and barren conditions, with a well-developed root system; thus it is an excellent tree for greening barren mountains, improving soil, and controlling soil erosion (Chen, 2008). At present, there are a lot of researches on the exploitation of fruit trees planted in saline-alkali land and the selection of saline-tolerant fruit tree resources, and great achievements have been made in the mechanism, physiology and heredity of saline-tolerant fruit trees. However, at the molecular level, there are few studies on salt-base stress of *C. humilis*, and no one has reported the response mechanism of the oxidation system of *C. humilis* under abiotic stress. In this study, *C. humilis* seedlings were subjected to alkali stress, and the physiological changes of plants before and after treatment and the changes of antioxidant levels with the extension of treatment time were studied. At the same time, combined transcriptome and non-targeted metabolomics techniques were used to identify differentially expressed genes (DEGs), differentially accumulated metabolites (DAMs), and their key pathways in the leaves of *C. humilis*. In addition, the *SOD* gene family of *C. humilis* was identified and analyzed, *WRKY* gene sensitive to alkali stress was screened from *C. humilis* through transcriptomic data, and W-box elements of *SOD* gene promoter region were cloned. The regulation of *WRKY* on *SOD* gene was verified by yeast single hybridization experiment. This study explored the mechanism of resistance to alkali stress in order to provide scientific theoretical basis for expanding the cultivation range and development and utilization of *C. humilis*.

## 2 Materials and methods

### 2.1 Plant material processing and transcriptome and metabolomics analysis

Two-year *C. humilis* saplings were used as experimental materials. The experimental materials were divided into control and alkali-treated groups for 4 and 8 days, with 3 biological replicates in each group. The control group was irrigated with distilled water and the experimental groups were irrigated with 200 mM sodium bicarbonate solution for one time every day. The leaves were collected after stress and then immediately frozen with liquid nitrogen and stored at minus 80-°C. In addition, transcriptome and metabolome analysis were performed on the seedlings of the control group and the alkali-treated group for 8 days. In accordance with the manufacturer’s guidelines, RNA extraction from tissues is executed utilizing conventional methodologies, subsequently accompanied by rigorous quality control of the RNA sample. This control primarily involves the utilization of an Agilent 2100 bioanalyzer for precise detection of RNA integrity. Following established protocols, RNA libraries are constructed employing samples that fulfill the necessary criteria (concentration exceeding 100 ng·uL^−1^). Following the creation of the library, preliminary measurement is conducted with a Qubit2.0 Fluorometer, and the library’s concentration is reduced to 1.5 ng/ul. Subsequently, the library’s insert size was measured using the Agilent 2100 bioanalyzer. subsequently, an Agilent 2100 A bioanalyzer is utilized to determine the library’s insert size. Once the insert sizes align with the anticipated dimensions, the library’s effective concentration is precisely quantified through qRT-PCR (with the effective concentration exceeding 1.5 nM). This rigorous quality control measure ensures the library’s integrity and efficacy. Effective concentration and targeted data volume requirements guide the pooling of different libraries, which is subsequently followed by Illumina sequencing. The FASTQ format is utilized for storing raw data, Following raw data filtering, sequencing error rate verification, and GC content distribution examination, clean reads suitable for subsequent analysis were obtained. Using the HISAT2 software, clean reads and reference genomes were swiftly and precisely compared to derive the genomic locations of reads on the reference genome (Mortazavi et al., 2008).

Samples were freeze-dried under vacuum and ground to powder. Fifty mg powder was weighed and added with 1,200 μL pre-cooled 70% methanol water internal standard extraction solution. The internal standard extraction solution was prepared by 1 mg standard solution dissolved in 1mL 70% methanol to prepare 1000 μg/mL standard mother solution, and the 1,000 μg/mL mother solution was further diluted with 70% methanol to prepare 250 μg/mL internal standard solution. Vortex once every 30 minutes, each lasting 30 seconds, a total of 6 vortices. After centrifugation, the supernatant was absorbed, the sample was filtered, and stored in the sample vial for subsequent analysis. Conditions for LC-MS: Hypersil Gold column (C18), column temperature: 40°C, flow rate: 0.2 mL/min, mobile phase A: 0.1% formic acid, mobile phase B: methanol, gradient elution procedures: 0~1.5 min: 2% B; 1.5~3.0 min: 85% B; 3.0~10.0 min: 100% B; 10~10.1 min: 2% B; 10.1~12.0 min: 2% B. Scanning range: m/z 100~1500; ESI source Settings were as follows: Spray voltage: ± 3.5kV (positive ion mode, negative ion mode); Sheath gas flow rate: 35 psi; Auxiliary gas flow rate: 10 L/min; Ion transfer tube temperature: 320°C; Ionic conductance Incoming RF level: 60; Auxiliary gas heater temperature: 350°C; The MS/MS secondary scan was a data-dependent scan. Secondary mass spectrometry was performed based on mzCloud, mzVault and Masslist databases. Python-3.5.0 software was used to analyze the mass spectrometry data to obtain the identification results of metabolites. Then, relative quantitative results of metabolites were obtained according to the relative peak area extracted after standardized processing. The differentiated metabolites were selected with VIP > 1.0, Fold Change FC > 1.5 or FC < 0.667, and significance level P-value< 0.05. Then, multivariate statistical analysis of metabolites was performed, including principal component analysis (PCA) and partial least square discriminant analysis (PLS-DA) and so on., to reveal the differences in metabolic patterns among different groups. Hierarchical clustering (HCA) and metabolite correlation analysis were used to reveal the relationships among samples and between metabolites and metabolites. Finally, the biological significance of metabolites was explained through functional analysis of metabolic pathways.

### 2.2 Identification and sequence analysis of *SOD* gene in *C. humilis*

BLASTP and HMM hidden Markov model was used to identify the *SOD* gene of *C. humilis*. For BLASTP, We used 8 *Arabidopsis* SOD amino acid sequences (AT1G08830.1/AtCSD1, AT2G28190.1/AtCSD2, AT5G18100.1/AtCSD3, AT4G25100.1/AtFSD1, AT5G51100.1/AtFSD2, AT1G51100.1/ATFSD2, AT4G25100.1 At5g2333.1/AtFSD3, AT3G10920.1/AtMSD1, AT3G56350.1/At00MSD2), e-value is set to 1e-5. The 8 amino acid sequences of ATSOD was downloaded from the TAIR Arabidopsis genome database. For further identification, Pfam, SMART and CDD websites conducted conserved domain validation on these obtained sequences and ultimately identified 9 members of the ChSOD family. 9 *SOD* gene members were renamed based on their location in chromosome and their types. The physical and chemical properties of ChSOD protein were analyzed by the online tool ExpasyProtParam (https://web.expasy.org/ProtParam) (**Supplementary Table S1**).

### 2.3 Phylogenetic analysis

A rootless phylogenetic tree was constructed using the neighborhood (NJ) algorithm of MEGA-11, and 1000 bootstrap were performed to study the phylogenetic relationship of *SOD* genes of *C. humilis* and *Arabidopsis thaliana*, *Setaria italica*, *Populus euphratica*, *Ricinus communis* and *Pyrus bretschneideri*. Retains the default value for other parameters. Finally, the phylogenetic tree was visualized using the Evolview online tool.

### 2.4 Gene structure and conservative motif analysis

The MEME tool (http://meme-suite.org/tools/meme) served to forecast the conserved motif in the *ChSOD* gene sequence. Pertinent settings are adjusted to achieve an ideal motif width ranging from 6 to 50, with a cap of 10 motifs. Using GSDS shows genetic structure (vers 2.0 http://gsds.cbi.pku.edu.cn/index.php-ion) check ChSOD genetic configuration. TBtools software demonstrated the configuration and preserved pattern of the *ChSOD* gene.

### 2.5 Prediction of cis-acting elements

Utilizing TBtools software’s Gtf/Gff3 sequences Extract program, two sequences, each 2,000 base pairs long, were extracted before the translation initiation site to serve as promoter regions. To delve deeper into the possible roles of cis-regulatory elements in the *ChSOD* gene, the cis-regulatory components of the promoter sequence underwent analysis via the PlantCare web tool, followed by a visual representation of the findings using the TBtools software’s Simple BioSequence Viewer (Chen et al., 2020).

### 2.6 Collinearity analysis

Use the circos program in TBtools to visualize intraspecific collinearity. The genome annotation files of *Malus pumila*, *Vitis vinifera*, *Arabidopsis thaliana* and *Oryza sativa* were retrieved from the Phytozome database. MCScanX software was used to examine the genome-wide collinearity between *C. humilis* and the other 4 species, and TBtools software was used to map the interspecific collinearity results.

### 2.7 Analysis of *ChSOD* gene expression

RNA was extracted and assessed for the integrity by 1.0% agarose gel electrophoresis. The RNA (2 mg per reaction) was reverse-transcripted into complementary DNA (cDNA) using Novizan first strand cDNA synthesis kit. The cDNA concentration was determined and 700 ng cDNA samples were taken from each well. Meanwhile, the specific primers of *ChSOD* gene were designed using Premier 3.0 software. and delivered to Harbin suitable biological company synthesis (**Supplementary Table S2**). qRT-PCR analysis was performed by qTOWER 2.2 real-time fluorescence quantification system (Analytik Jena, Jena, Germany). The actin gene from *C. humilis* served as the control in this study. This method was replicated thrice for every cDNA specimen. The 2^−ΔΔCT^ technique was employed to calculate period threshold (CT) data from qRT-PCR, aiming to ascertain gene expression levels. The analysis of expression data was conducted utilizing Graphpad Prism9 (Liu et al., 2018). At the same time, we used transcriptome data to study the expression changes of *ChSOD* gene during salt stress, and used log2 transformation (FPKM + 1) value to generate tissue-specific expression profile heat maps. Finally, TBtools was used to visualize the *SOD* gene expression level.

### 2.8 Yeast single hybridization experiment

The yeast strain EGY48 was used in yeast single hybridization, and the carriers were pLacZ-2u and pJG4-5. The yeast was transformed according to the LiCI-PEG method in the Yeast Manual (Clontech), and the yeast single hybridization was detected in the chromogenic SD medium lacking Ura and Trp and adding X-gal. Using https://www.yeasen.com/hieff-clone/homologous primer design, carrier, fragment sequences uploaded to the website, and select the appropriate enzyme loci, the homologous can generate sequence primers (**Supplementary Table S3**). In addition, vector primers pLacZ-F/R and pJG4-F/R were designed on SnapGene software for positive cloning identification and sequencing. The target gene ChWRKY was amplified with homologous arms on both sides of the pJG4–5 cleavage site by using the cDNA of *C. humilis* as template and PJG4-5-Chwrky-F/R as primer. Using Euclidean DNA as the template and pLacZ-ChSOD PRO-F/R as the primer, the target gene ChSOD pro with homologous arms on both sides of pLacZ-2u cleavage site was amplified. The obtained target fragment was inserted into the corresponding enzyme digestion vector.

### 2.9 Oxidation index determination

The determination methods were carried out according to the instructions of SOD, CAT, POD enzyme activity kit and MDA content determination kit (Suzhou Gris Biotechnology Co., LTD.).

### 2.10 Analysis of potential protein interaction

The SOD protein interaction network was constructed by STRING 11.0 (https://string-db.org/cgi/input.pl). Set the network edge to confidence, set the parameter to medium confidence parameter (0.400), and display no more than 10 interaction factors.

## 3 Results

### 3.1 Transcriptome analysis

**Figure 1:**
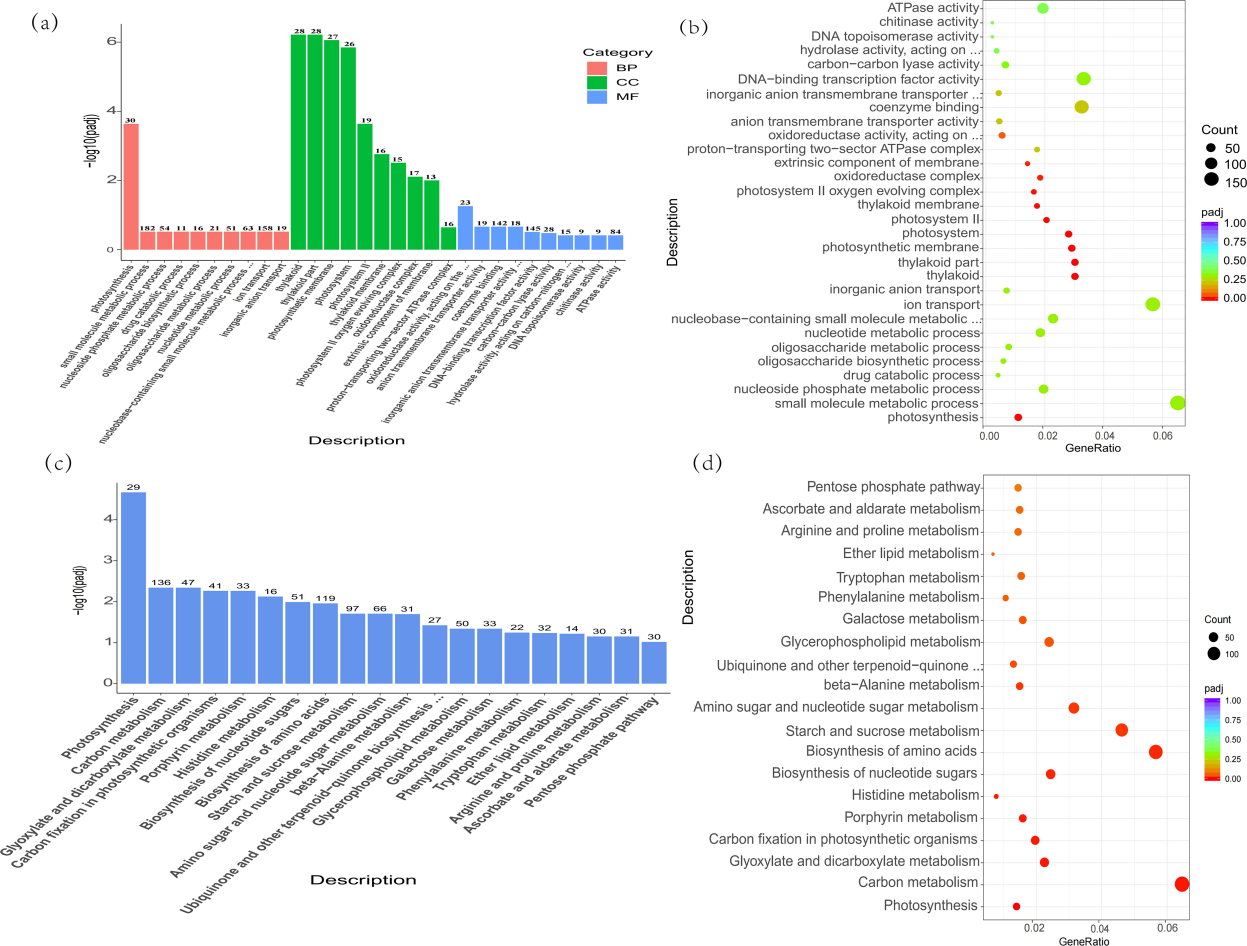
**(a)** The GO enrichment analysis histogram, the horizontal axis denotes the GO Term and the vertical axis the enrichment level of the GO Term, denoted by -log10 (padj). A greater value holds increased significance. The column indicates the count of distinct genes incorporated into the pathway. Varied hues signify diverse functional classifications. **(b)** The scatter plot for GO enrichment analysis, the horizontal axis represents the proportion of differential genes labeled to GO Term relative to the overall differential gene count, while the vertical axis indicates GO Term, the dot size indicates the quantity of genes linked to GO Term, with the shift from red to purple signifying the importance of enrichment. **(c)** Graphical representation of KEGG enrichment studies. Within the chart, the horizontal axis represents the KEGG pathway, while the vertical axis indicates the degree of pathway enhancement. As the value increases, its significance escalates. **(d)** In the KEGG enrichment point diagram, the horizontal axis represents the proportion of differential genes marked in the KEGG pathway relative to the overall differential gene count, while the vertical axis denotes the KEGG pathway, the magnitude of Points indicate the count of genes marked in the KEGG pathway, while the shift from red to purple signifies the importance of enrichment.

Illustrated in **Figure 1**, when subjected to alkali stress, *C. humilis* yielded 9,602 differentially expressed genes (DEGs), with 4,674 genes experiencing down-regulation and 4,928 genes up-regulation. In order to delve deeper into the roles of these DEGs, they are categorized into three primary groups according to the GO database, encompassing biological processes, cellular elements, and molecular activities. Among them, the biological processes under alkali stress were mainly photosynthesis (GO:0015979), small molecule metabolism (GO:0044281), nucleotide metabolism (0009117), oligosaccharide metabolism (GO:0009311), oligosaccharide biosynthesis (GO:0009312), and so on. The results showed that *the stressed saplings* had extensive metabolic activity under alkali stress. The cell components were mainly chloroplast (GO:0015979), photosynthetic membrane (GO:0034357), photosystem II oxygen-generating complex (GO:0009654), REDOX enzyme complex (GO:1990204), and so on. The molecular functions mainly focus on REDOX enzyme activity: acting on CH-CH group donor (GO:0016627), anion transmembrane transporter activity (GO:0008509), DNA-binding transcription factor activity (GO:0003700), ATPase activity (GO:0016887), and so on.

In addition, based on KEGG database, the functions of DEGs in *C. humilis* are annotated. It was found that DEGs under alkali stress were concentrated in photosynthesis (ath00195), carbon metabolism (ath01200), histidine metabolism (ath00340), amino acid biosynthesis (ath01230), starch and sucrose metabolism (ath00500), et.

### 3.2 Metabolome analysis

**Figure 2:**
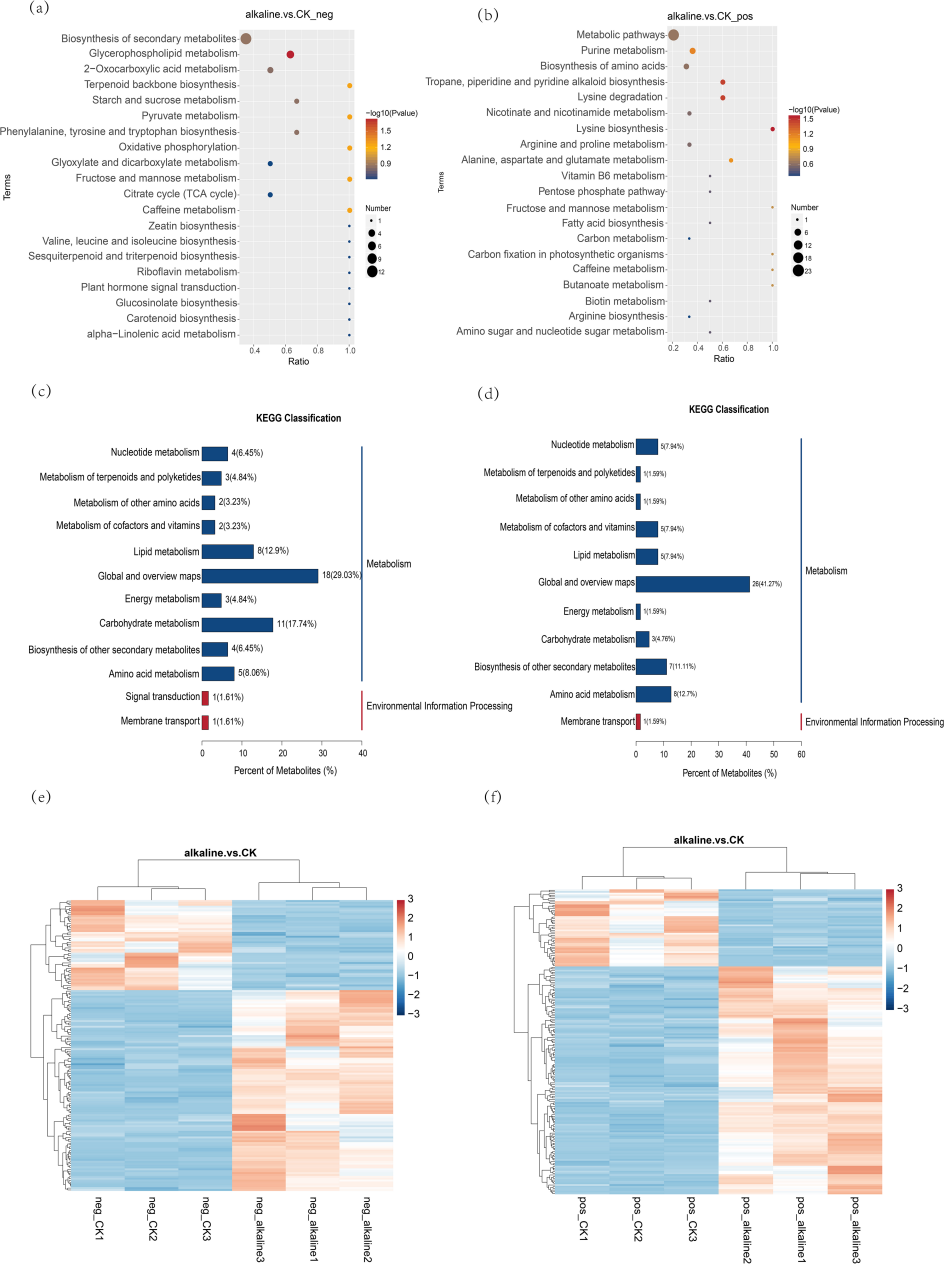
**(a, b)** In the KEGG enrichment bubble diagram, the horizontal axis is defined as x/y, representing the ratio of differential metabolites in a specific metabolic pathway to the pathway’s total identified metabolites; a greater value indicates a higher value, the higher the concentration of differential metabolites in the pathway. The hue of the dots signifies the hypergeometric test’s P-value, and a lower value indicates greater reliability and statistical significance of the test. The dot’s dimensions indicate the count of distinct metabolites present in the respective The pathway’s complexity increases with the size of the point, leading to a higher variety of metabolites within it. Within this group, **(a)** illustrates the negative ion mode, while **(b)** depicts the positive ion mode. **(c, d)** Diagram of KEGG categorization for varying metabolites. Within the chart, the horizontal axis signifies the proportion of metabolites marked in a KEGG Pathway across all such annotated metabolites. The right side of the vertical coordinate is the KEGG Pathway primary classification, and the left side is the KEGG pathway secondary classification. Among them, **(c)** represents the negative ion mode, and **(d)** represents the positive ion mode. **(e, f)** Heat map for various metabolite clusters, with vertical representing the sample group and horizontal the metabolite cluster, indicating greater similarity as the cluster branch length decreases. The clustering of metabolite content across different groups is observable. In this context, **(e)** illustrates the negative ion mode, while **(f)** depicts the positive ion mode.

As shown in **Figure 2**, under alkali stress, 182 different metabolites were detected in the positive ion mode, among which 136 metabolites were up-regulated and 46 metabolites were down-regulated. In the negative ion mode, 155 different metabolites were detected, among which 107 metabolites were up-regulated and 48 metabolites were down-regulated. Mapping these metabolites to the KEGG pathway, we found that they were mainly concentrated in nucleotide metabolism, terpenoid and polyketone metabolism, cofactor and vitamin metabolism, lipid metabolism, energy metabolism, carbohydrate metabolism, other secondary metabolites metabolism, and amino acid metabolism, indicating that alkali stress can affect the changes of multiple metabolites and metabolic pathways of *C. humili*.

### 3.3 Screening of ChWRKY gene

**Figure 3:**
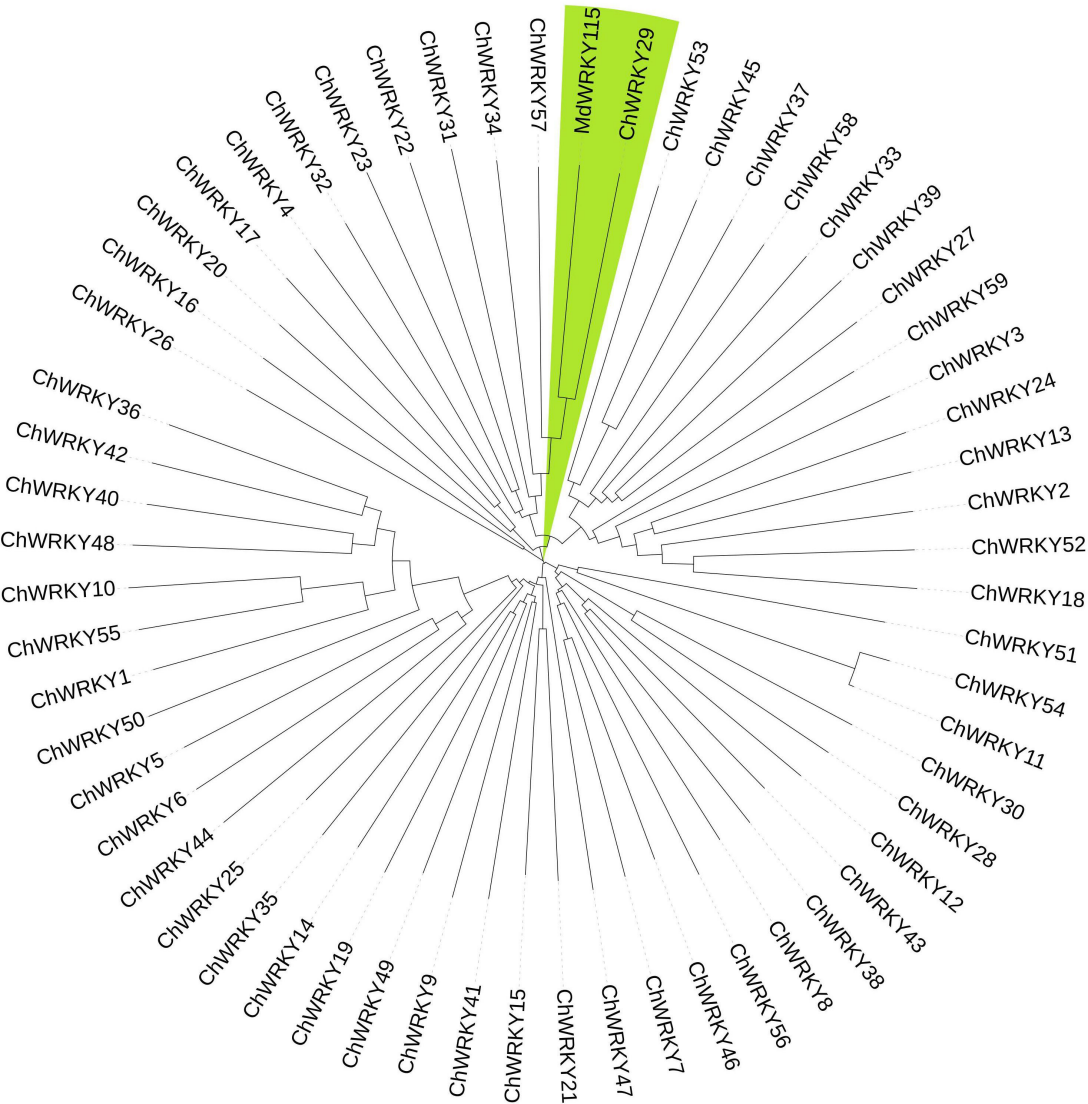
Phylogenetic tree of ChWRKY and MdWRKY115.

A previous research indicates that WRKY TFs control plant reactions to non-living stressors by attaching to gene promoters with W-box cis-elements and collaborating with various proteins to create intricate control networks (Hu et al., 2012; Jiang and Yu, 2016; Rushton et al., 2010). *MdWRKY115* plays a crucial role in controlling osmotic and drought stress resistance in *Malus pumila*. *MdWRKY115* has the ability to attach to the W-box segment of the downstream gene promoter, controlling its expression and contributing to osmotic pressure and drought stress (Dong et al., 2024). *C. humilis* and *M. pumila* belong to the rose family. In this study, ChWRKY and MdWRKY115 were used to construct phylogenetic tree (**Figure 3**), and *ChWRKY29* was screened out. At the same time, *ChWRKY34*, which was closely related to *ChWRKY29*, was screened out according to differential expression of genes under alkali stress. They were used to study the regulation of SOD gene under alkali stress.

### 3.4 Identification and physicochemical properties of *ChSOD* gene family

By removing untargeted, overlapping, or truncated protein sequences, we identified nine *SOD* genes in the *C. humilis*. Three conserved *SOD* domains PF00080, PF00081 and PF02777 were found in all the 9 *SOD*s identified in *C. humilis*. Both Fe-SODs and Mn-SODs possess domains for iron/manganese superoxide dismutase, α-hairpin (Pfam: 00081), and C-terminal (Pfam: 02777). Cu/Zn-SOD possesses a domain for copper/zinc superoxide dismutase (Pfam: 00080). Furthermore, the presence of the heavy metal domain (PF00403) was noted in ChCSD5.

The protein encoded by *ChSOD* gene ranges in length from 152 to 738 amino acid residues, molecular weight from 15.42 to 81.21 kDa, and pI value from 5.14 to 8.84. It shows that the protein changes from acidic to basic.

### 3.5 Phylogenetic analysis and chromosome localization

**Figure 4:**
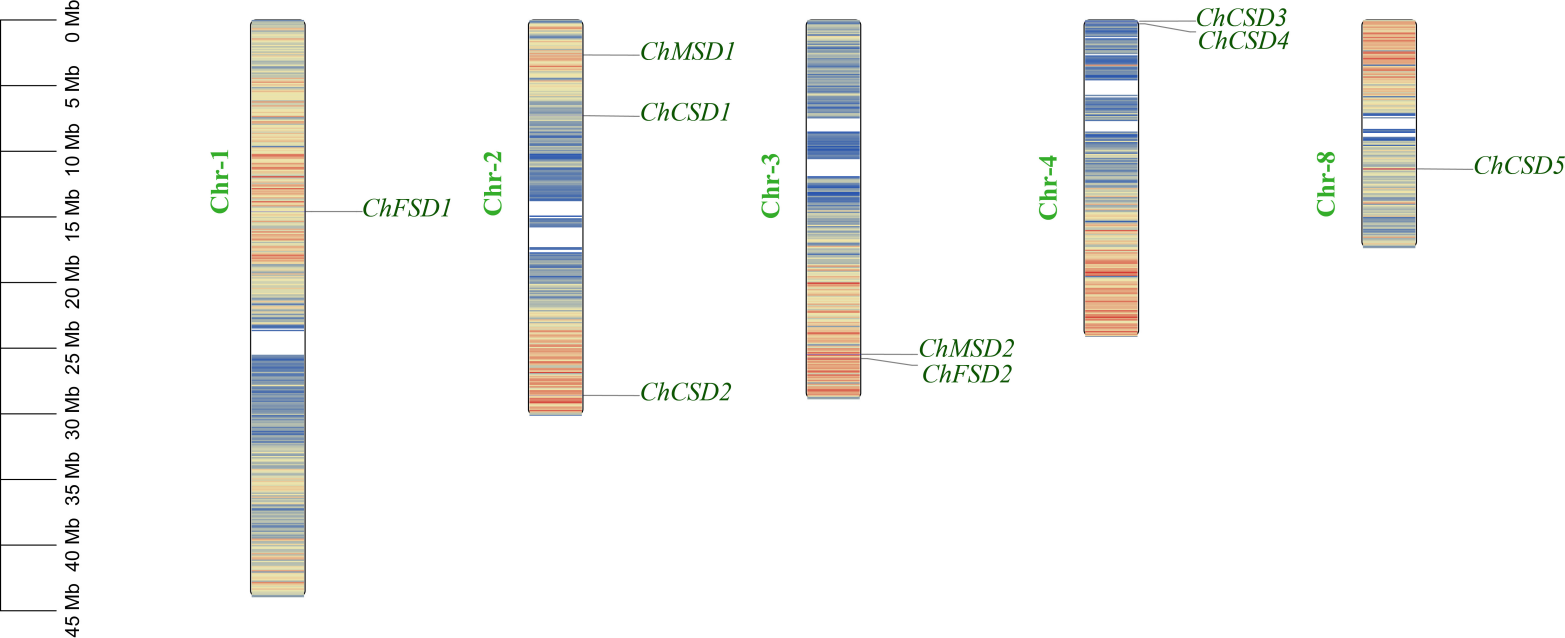
*ChSOD* chromosome mapping.

**Figure 5:**
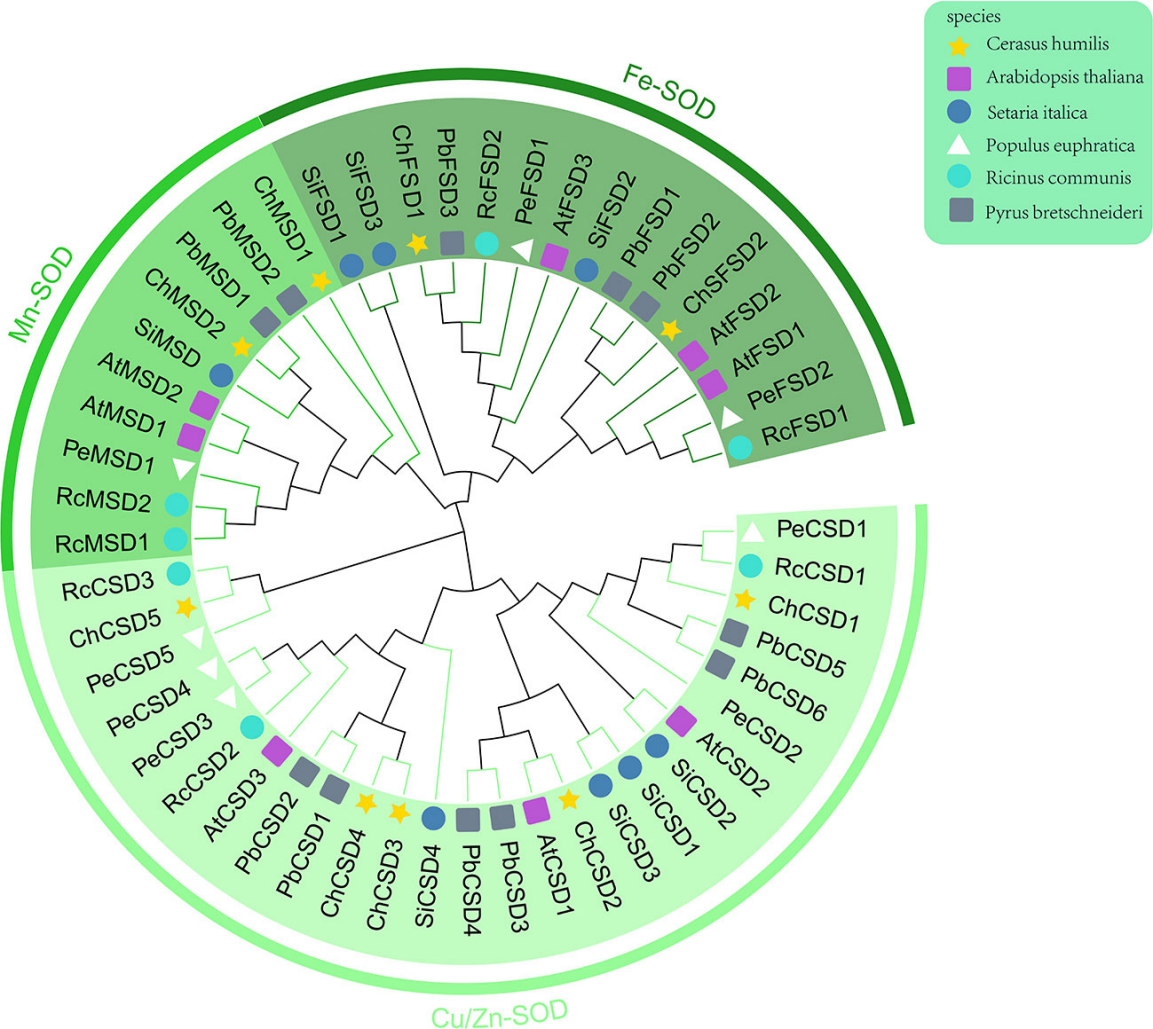
Phylogenetic tree of SOD protein in 6 species of *C. humili*, *Arabidopsis thaliana*, *Setaria italica*, *Populus euphratica*, *Ricinus communis* and *Pyrus bretschneideri*.

Firstly, according to chromosome localization and subgroup classification, we renamed the members of the *SOD* gene family (**Figure 4**). As can be seen from **Figure 4**, *ChSODs* were randomly distributed in five chromosomes of *C. humilis*, among which the number of Chr-2 was the largest. In order to study the relationship and classification of members of the *SOD* gene family, we constructed an evolutionary tree containing 51 protein sequence (**Figure 5**), They came from six species: *C. humili*, *A. thaliana*, *S. italica*, *P. euphratica*, *R. communis* and *P. bretschneideri*. According to the classification of *SOD* genes in *A. thaliana* and *S. italica*, the model plants were divided into three subgroups: Fe-SOD, Mn-SOD and Cu/Zn-SOD, in which Cu/Zn-SOD contained 5 *ChCSDs*, Mn-SOD contained 2 *ChMSDs*, and Fe-SOD contained 2 *ChFSDs*. ChSOD protein sequences contribute to all subgroups. These results suggest that events of gene loss or acquisition may have occurred throughout evolution. The gain and loss of specific *SOD* gene members led to functional differentiation. *C. humilis* and *P. bretschneideri* genes were closely clustered compared to the other species, suggesting that *C. humilis* and *P. bretschneideri* genes were more similar in the other four species.

### 3.6 Gene structure and conserved motif analysis

**Figure 6:**
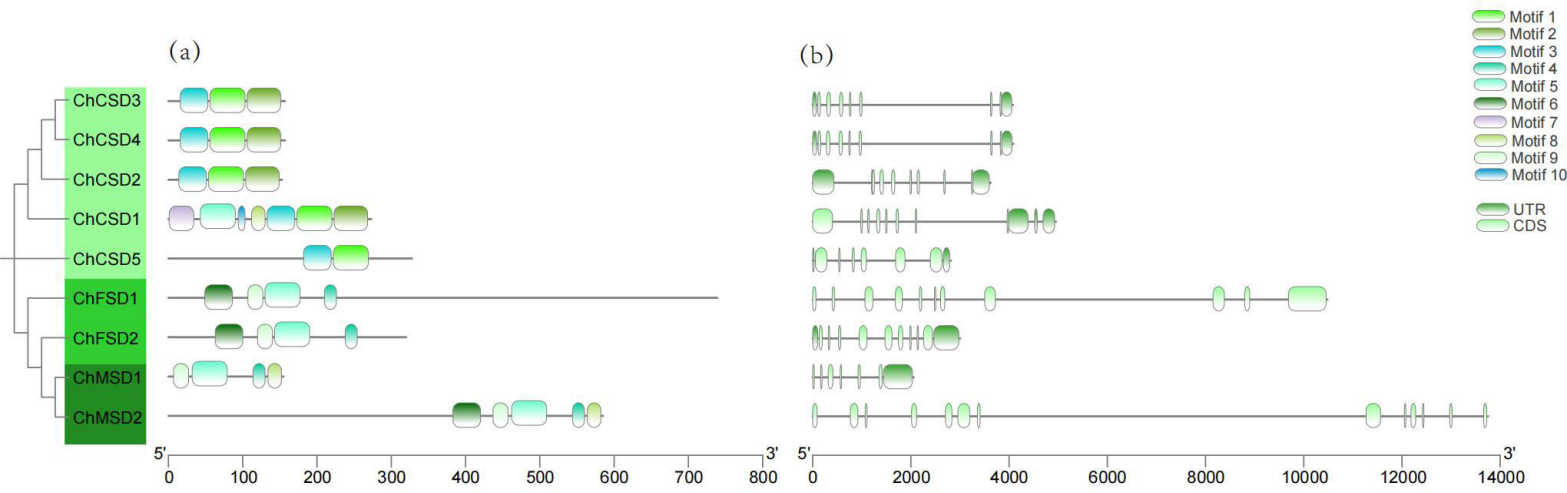
**(a)** *ChSOD* conserved motif, **(b)** *ChSOD* gene structure, black lines represent introns.

Further conservative motif examination uncovered the structural features of *ChSOD*. A total of ten preserved motifs were pinpointed (**Figure 6a**), Besides the lack of motif 2 in *ChCSD5*, motifs 1, 2, and 3 were also found in 4 Cu/Zn-SODs, while motifs 5 and 9 were present in 4 Fe-SODs and Mn-SODs. The findings suggest a significant resemblance in motif makeup among members within the same subfamily. Motifs 1, 3, 4 and 9 constitute the key functional domains of the above *SOD* genes. Motifs 1 and 3 are associated with the Cu/Zn-SOD domain (PF00080) and have only been identified in members of the Cu/Zn-SOD subfamily. Motifs 4 and 9 correspond to the Fe/Mn-SOD α-hairpin domain (PF00081) and Fe/Mn-SOD C-terminal domain (PF02777), respectively, and have only been identified in Fe/Mn-SODs.

The quantity of introns and the configuration of genes frequently correlate with the evolutionary patterns of members in the gene family (Zhang et al., 2021). We also examined the intron-exon structure of *ChSOD* members (**Figure 6b**). These members have 5–12 exons, among which *ChSOD5* has the lowest introns and ChMSD2 has the most introns. Even though most *ChSOD* genes are closely related, the size and distribution of exons/introns are still different. The diversity of gene structure helps to understand the long-term evolutionary mechanism of polygenic families.

### 3.7 Cis-acting element prediction

**Figure 7:**
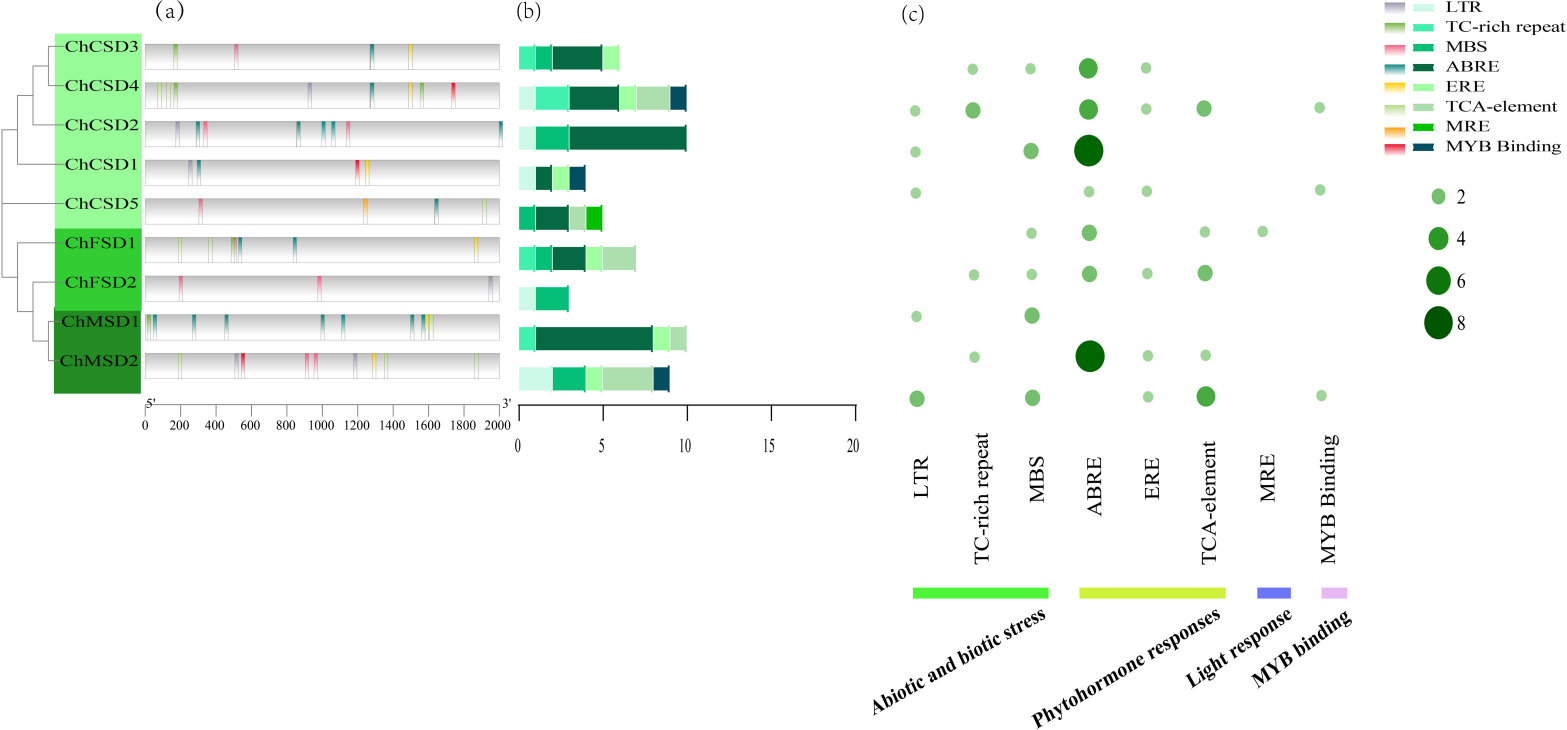
**(a)** Cis-acting element distribution, **(b)** The number of 8 cis-acting elements in each *ChSOD* gene, **(c)** The distribution of 8 cis-acting elements in *ChSOD* gene.

To further understand the regulatory network that controls expression, we identified the type and number of cis-elements in the *ChSODs* promoter region. Eight typical components were selected for analysis (**Figure 7**), They contain three hormone regulatory elements, namely EREs, ABREs and TCA elements, which are related to ethylene, Abscisic acid and salicylic acid response, respectively. The abundance of these elements suggests that plant hormones may have regulatory effects on *ChSOD* gene expression. Three regulatory components of stress response were also found in the *ChSOD* promoter region, including mbs, LTRs and TC-rich repeats, which were related to abiotic defense and stress response, and MYB-binding elements were also closely related to stress. A considerable number of plant response cis-elements were detected in the promoter region of *SOD* gene family in *C. humili*, indicating that the cis-elements of *SOD* promoter had a positive response to abiotic stress. However, different from the previous identification in rosaceae, the number of photoresponsive elements in the *ChSOD* promoter region is small, which may indicate that the *SOD* gene in *C. humili* plays a weak role in light regulation (Li et al., 2021).

### 3.8 Collinearity analysis

**Figure 8:**
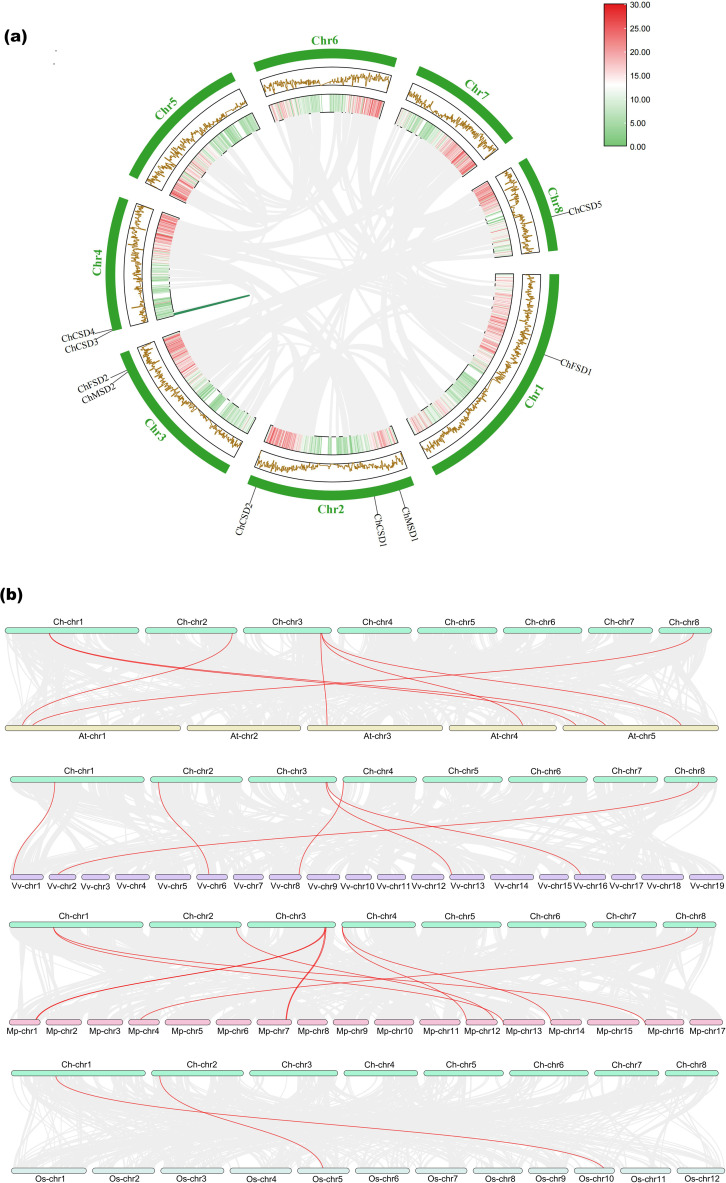
**(a)** Intraspecific collinearity analysis of *ChSOD* gene, **(b)** Interspecific collinearity analysis of *ChSOD* gene and *SOD* gene of 4 other species (*Malus pumila*, *Vitis vinifera*, *Arabidopsis thaliana* and *Oryza sativa*).

Gene replication plays an important role in the organic evolution and functional diversity of plants (Xu Zhang et al., 2022). Gene duplication analysis showed that *ChCSD4* and *ChCSD5* had fragment duplication based on the chromosome distribution of *ChSOD* gene, while there was no tandem replication between *ChSOD* genes (**Figure 8a**). It is suggested that fragment repetition may be the main driving force for the evolution of *SOD* gene family in *C. humili*.

The results of interspecific collinearity analysis showed that homologous *SOD* gene existed between *C. humili* and other 4 species (*M. pumila*, *V. vinifera*, *A. thaliana* and *O. sativa*) (**Figure 8b**). Among them, there were 7 pairs of homologous *SOD* genes with *A. thaliana*, 8 pairs with *M. pumila*, 6 pairs with *V. vinifera* and 2 pairs identified with *O. sativa*. *ChCSD1* and *ChCSD4* were not localized to any of the four co-strand blocks of species with the *SOD* gene, suggesting that these chromosomes underwent extensive rearrangement and fusion, possibly leading to selective gene loss (Zheng et al., 2023). In addition, it can be seen that among the four species, *C. humili* is the most closely related to *M. pumila*.

### 3.9 Analysis of *ChSOD* gene expression

**Figure 9:**
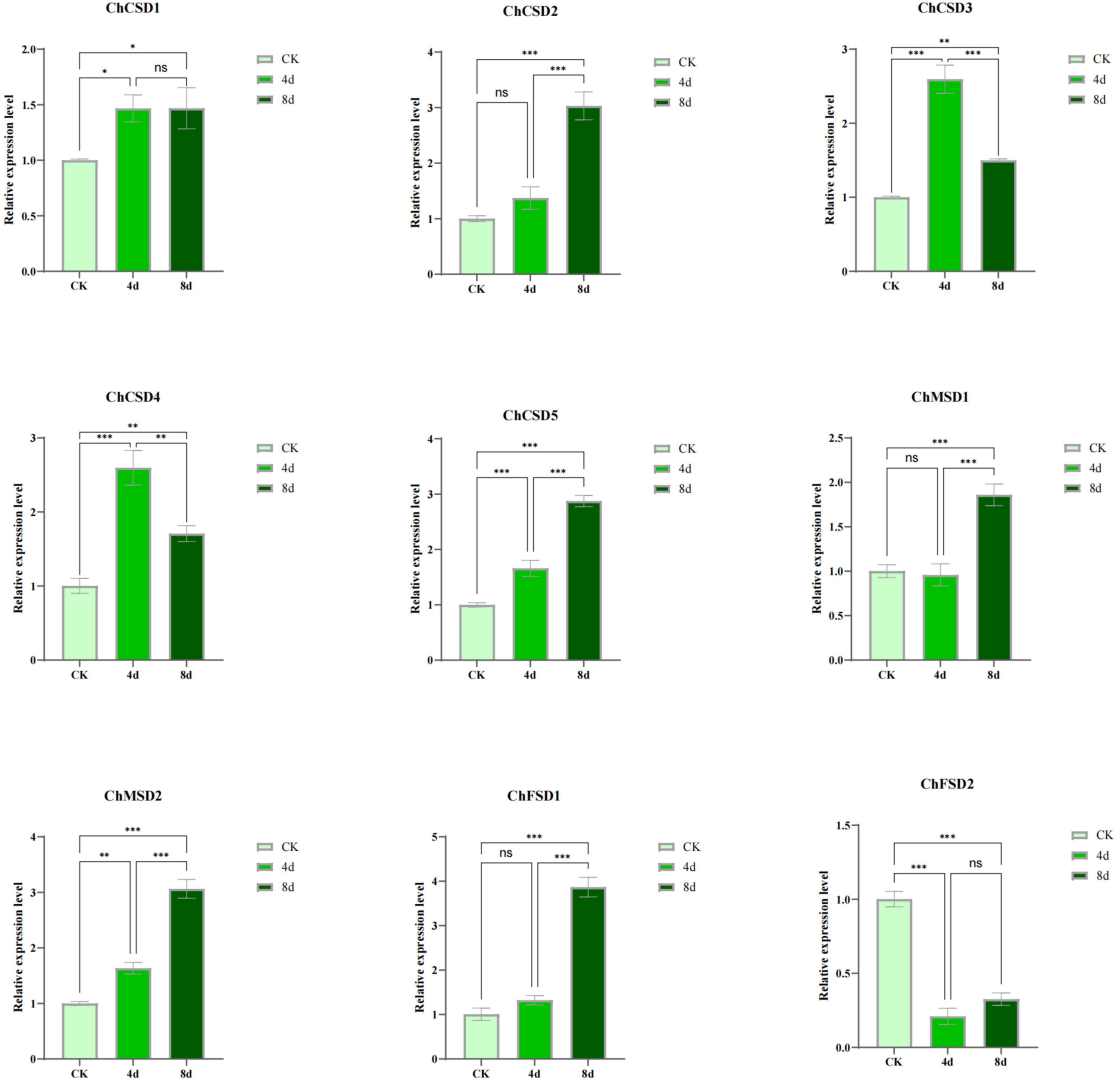
*ChSOD* gene qPCR analysis, horizontal coordinate was different groups, followed by control and alkali-treated groups for 4 and 8 days. the horizontal coordinate indicates relative gene expression. (p* < 0.05; p** < 0.01; p*** < 0.001).

**Figure 10:**
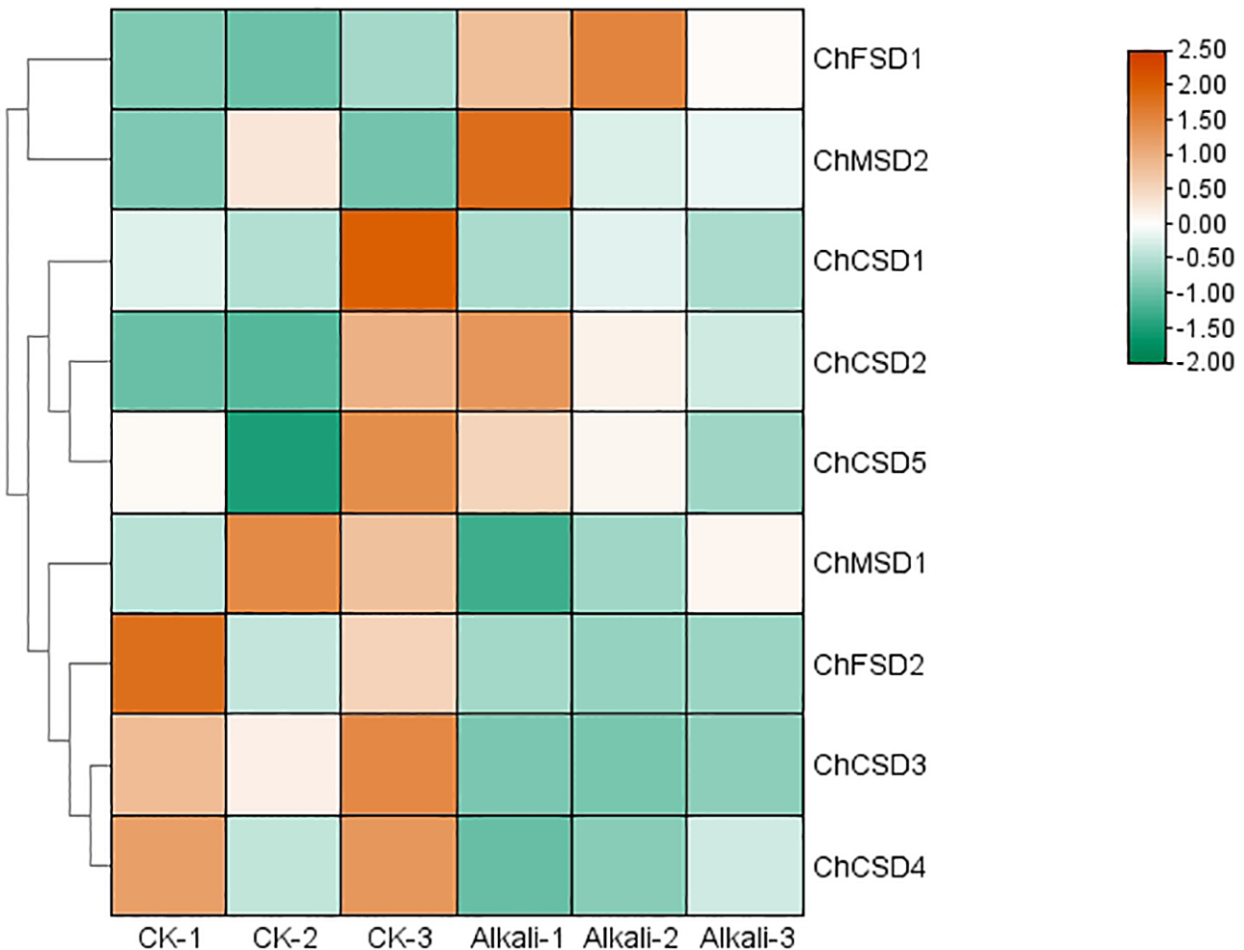
*ChSOD* gene transcriptome heat map, horizontal coordinate is different groups, control group and alkali treatment group for 8 days in order, three parallel in each group, vertical coordinate is gene, color from green to red indicates the expression level from low to high.

In order to study the response of *SOD* to alkali stress, we used qPCR and transcriptome heat map to study the expression pattern of *C. humili* under alkali stress (**Figure 9**, **10**). The results showed that all *ChSOD* genes were significantly induced or inhibited under alkali stress. With the prolongation of alkali stress time, the expression levels of *ChCSD1*, *ChCSD2*, *ChCSD5*, *ChMSD1*, *ChMSD2*, and *ChFSD1* genes increased, the expression level of *ChFSD2* gene decreased, and the expression levels of *ChCSD3* and *ChCSD4* genes first increased and then decreased. The transcriptional data showed that *ChFSD1*, *ChCSD2* and *ChMSD2* were significantly up-regulated and the rest were down-regulated under alkali stress. These results all indicated that *ChSOD* played an important role in the plant resistance to alkali stress, and homologous genes showed different response patterns.

### 3.10 Physiological index analysis

**Figure 11:**
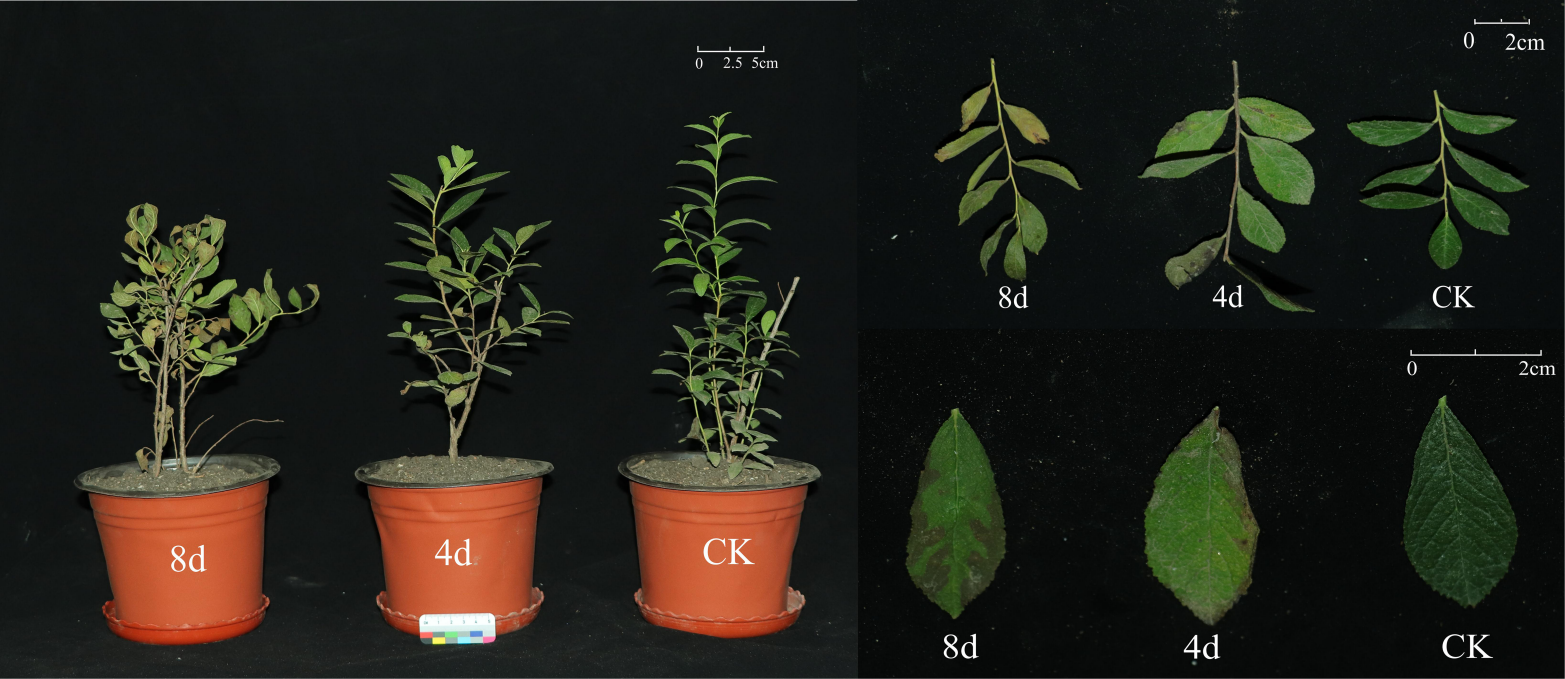
Control group and alkali treatment for 4 days, 8 days of *C. humili* plant.

As shown in the figure, it can be observed that with the extended alkali treatment, the plant leaves appeared obvious dry and yellowing, or even wilting and falling off, and the brown area on the edge of the leaves gradually increased (**Figure 11**).

**Figure 12:**
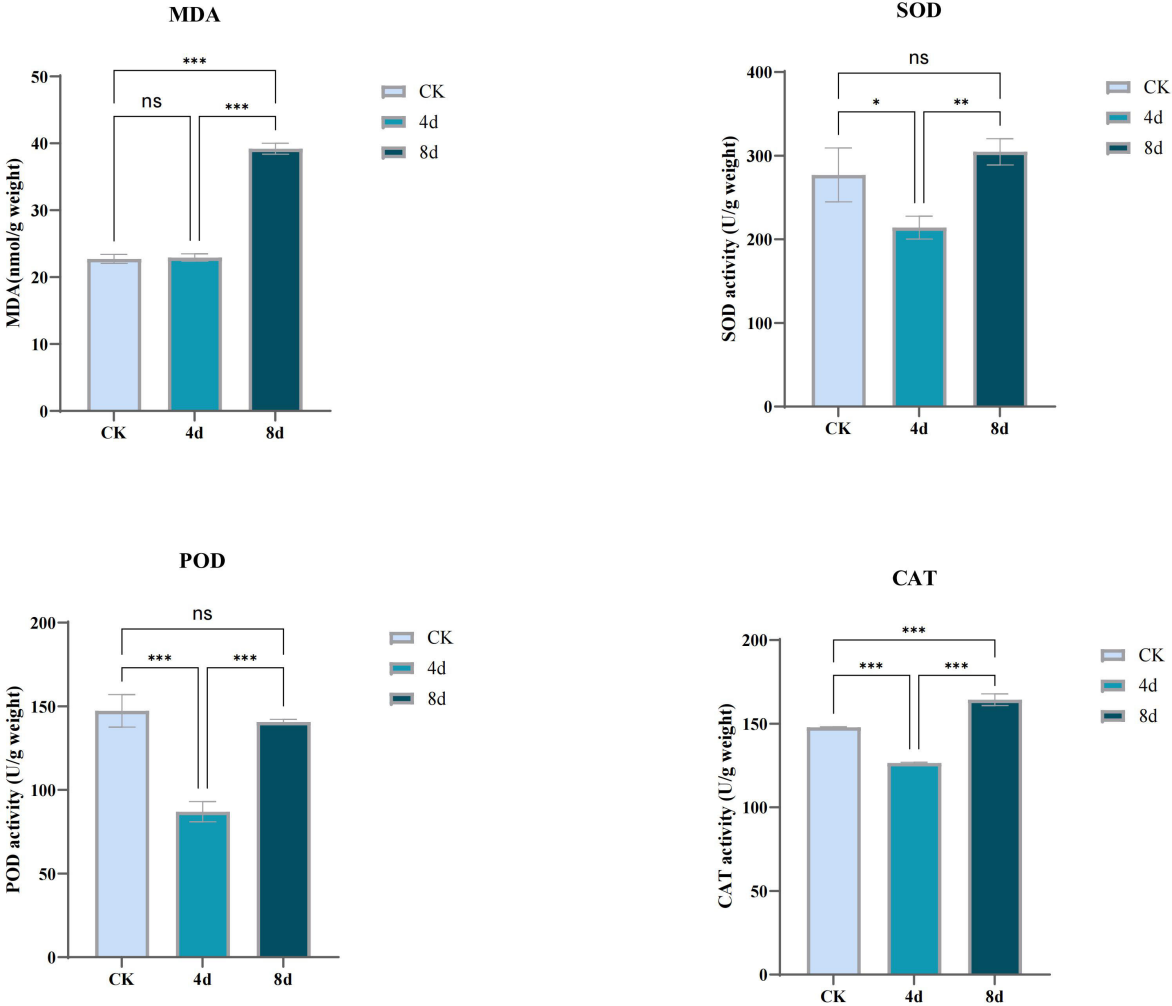
Determination of activity of antioxidant enzymes (SOD, POD, CAT) and content of MDA in *C. humili* treated with alkali. (p* < 0.05; p** < 0.01; p*** < 0.001).

The antioxidant levels of *C. humili* plants under alkali stress were determined in this study (**Figure 12**). Malondialdehyde (MDA) is the final decomposition product of lipid peroxidation of cell membrane, and its content can reflect the level of membrane peroxidation and the degree of damage. The content of MDA was stable after 4 days of alkali treatment, and significantly increased after 8 days of alkali treatment. The results indicated that the damage degree of plants increased with the extension of alkali stress time.

When subjected to stress, plants will accumulate reactive oxygen species (ROS), which will produce oxidative stress on plants and inhibit plant growth. In order to resist the negative effects of stress, plants activate the antioxidant enzyme system in the body or increase the amount of antioxidant substances to eliminate ROS. SOD, POD and CAT belong to antioxidant enzymes, and the determination of the activity of these enzymes is helpful to understand the effect of alkali stress on plants. With the extension of treatment time, the activities of the three antioxidant enzymes first decreased and then increased, indicating that MDA accumulation and electrolyte permeability increased *in vivo* after treatment for 8 days, and the antioxidant mechanism was activated.

### 3.11 Yeast single hybridization experiment

**Figure 13:**
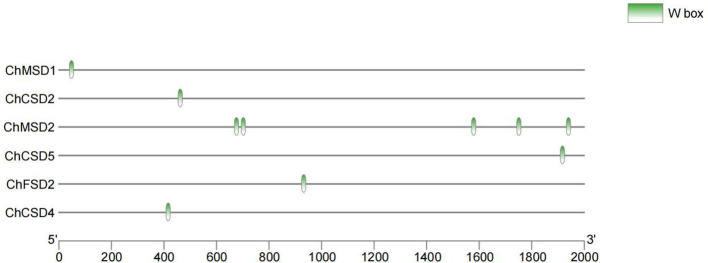
Distribution of W-box elements in *ChSOD* gene promoter region.

**Figure 14:**
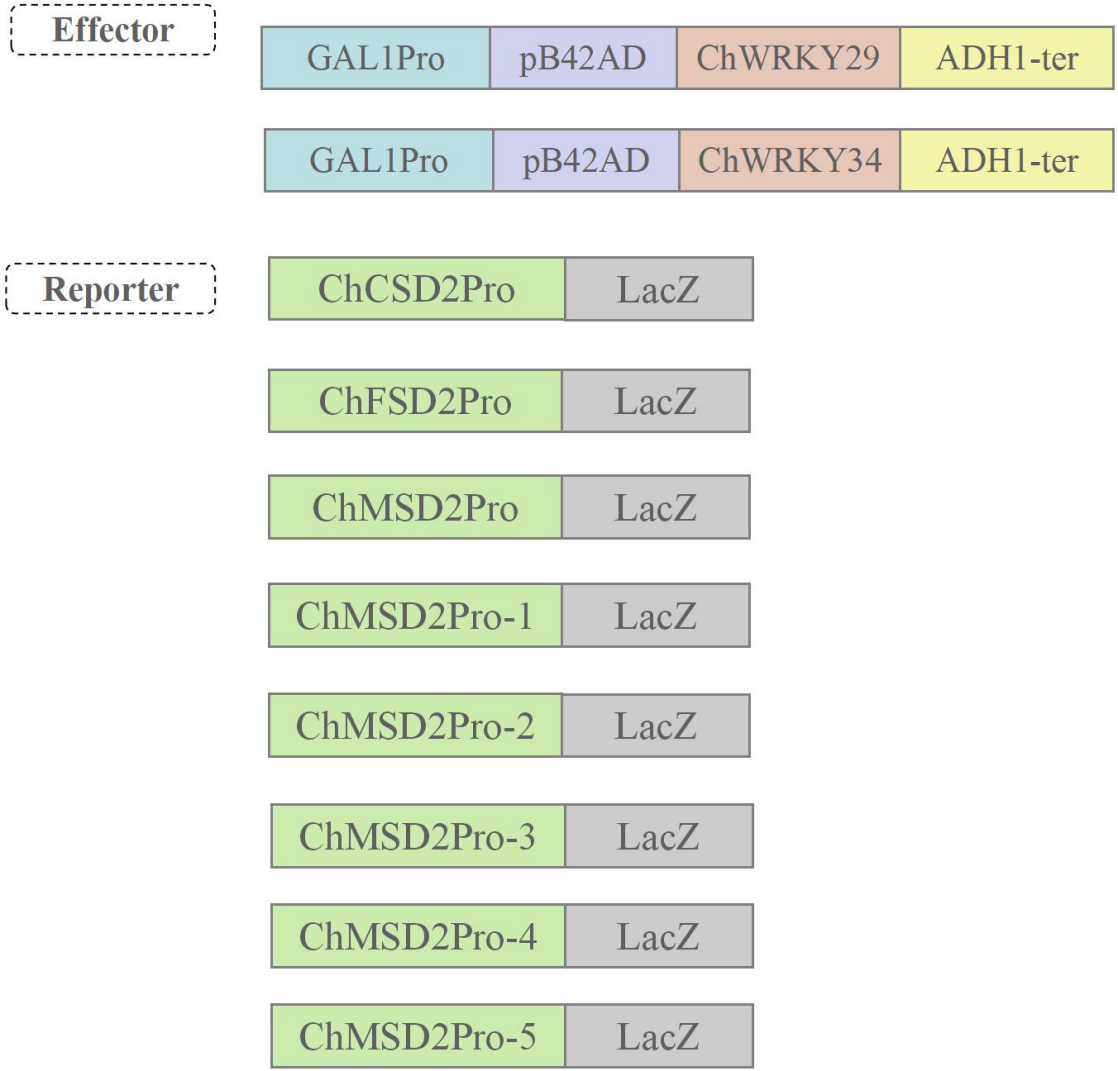
Construction diagram of yeast single hybrid plasmid.

**Figure 15:**
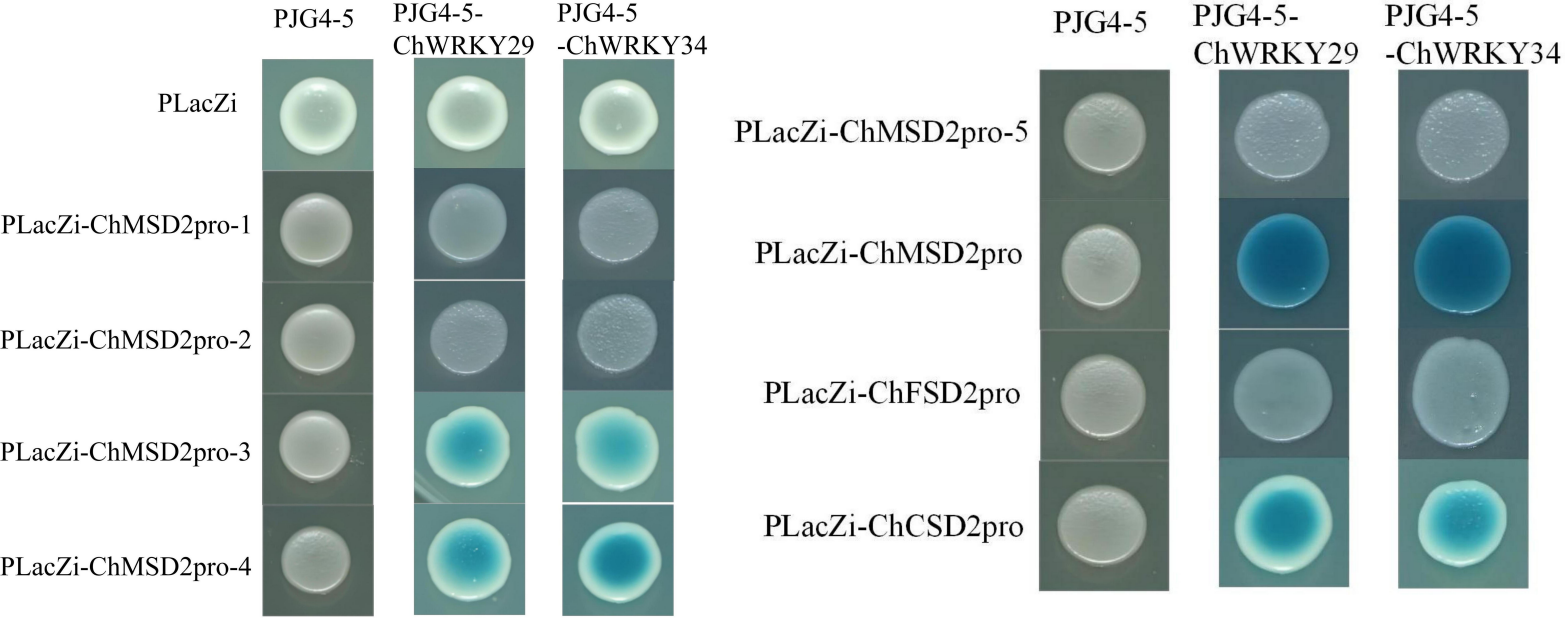
*ChWRKY29*, *ChWRKY34* and *ChMSD2*, *ChFSD2*, *ChCSD2* yeast single hybridization test results.

Multiple studies have demonstrated that WRKY regulates the expression of target genes by directly binding to W-box elements (Phukan et al., 2016; Ülker and Somssich, 2004). In order to study the regulatory mode of *ChWRKY29* and *ChWRKY34* on *ChSODs*, we performed Y1H assay (**Figures 13**–**15**). First, we predict w-box elements in the *ChSODs* promoter region (**Figure 13**). Three *ChSOD* members were randomly selected to co-transform yeast strain EGY48 with ChWRKY29 and ChWRKY34 (in which ChCSD2 had five W-box promoter, all of which were cloned in sections). To detect the binding of *ChWRKY* to the W-box site of *ChSOD* gene *in vitro*. The results showed that *ChWRKY29* and *ChWRKY34* interacted with W-box fragment on *ChCSD2* and *ChMSD2*, respectively, but did not interact with *ChFSD2* in yeast cells. Among the five sites in *ChCSD2*, only the third and fourth sites interacted with each other.

### 3.12 Analysis of potential protein interaction

**Figure 16:**
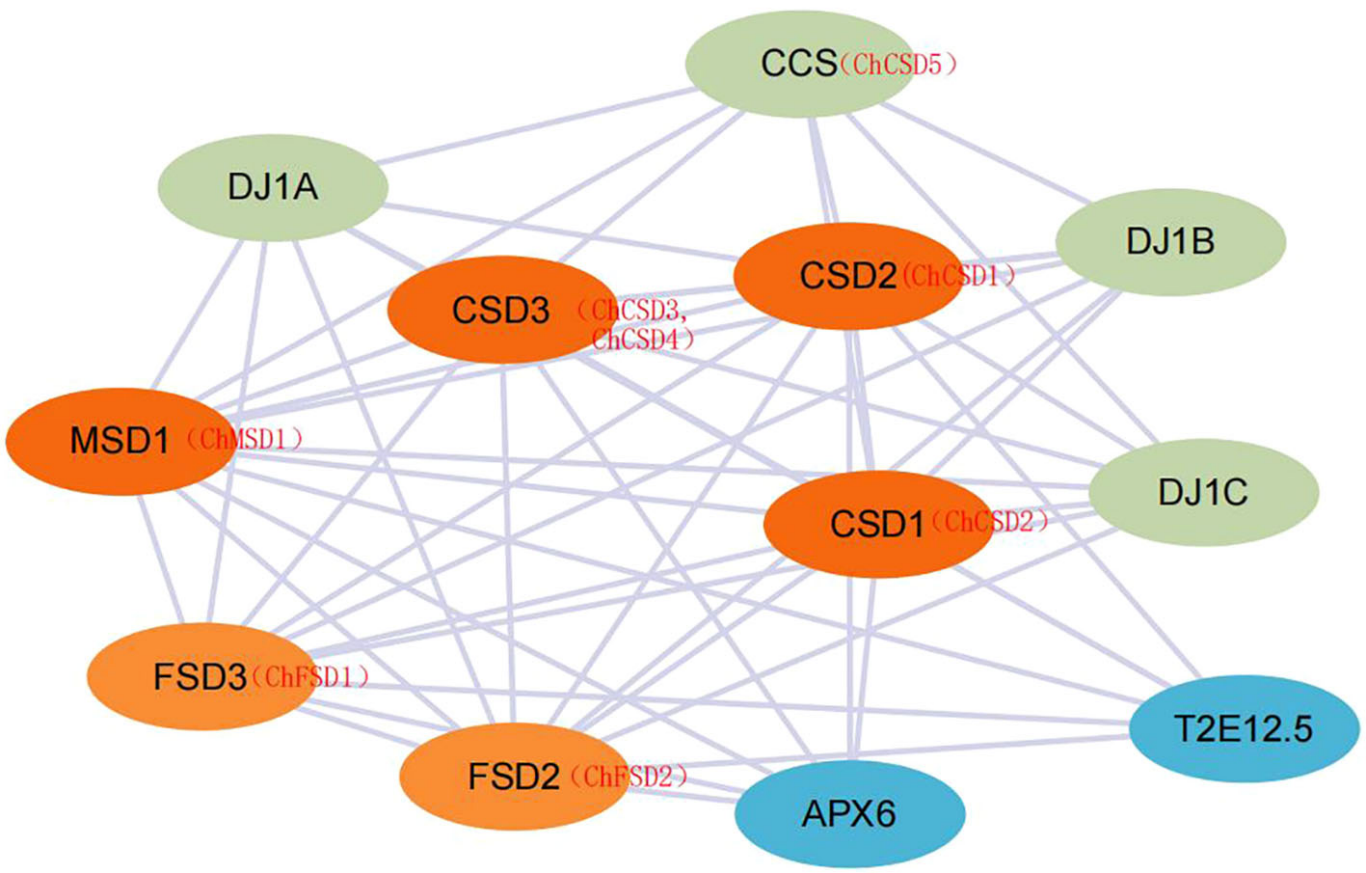
ChSOD protein interaction prediction network.

To explore potential regulatory roles between ChSOD proteins, a protein interaction network was constructed using the STRING database (**Figure 16**). The results showed that 8 ChSOD proteins interacted with each other, while ChMSD2 did not interact with any ChSOD protein, and ChCSD1, ChCSD2 and ChMSD1 interacting proteins were the most, interacting with all 7 ChSOD proteins. Prediction of ChSOD protein interaction networks may help to understand functional associations between these proteins.

## 4 Discussion

The primary abolic stressor impeding agricultural output is the saline-alkali stress (Gong et al., 2013; Nishiuchi et al., 2010). In saline-alkali environments, plants adjust to environmental shifts by triggering various stress reactions, aiming to safeguard cells and sustain growth. Such reactions necessitate the articulation of gene responses to stress governed by the TF network (Mizoi et al., 2012; Wang et al., 2021). The WRKY transcription factors are linked to a variety of abolic stress reactions. The heightened expression of *TaWRKY55* in *Triticum aestivum* showed sensitivity to stress from saline and alkali environments (Wei et al., 2024). The *SlWRKY81* variant enhanced *Solanum lycopersicum*’s resilience to salt-alkali stress by boosting antioxidant energy, root function, and proline levels, while reducing malondialdehyde levels (Shang et al., 2024). However, the molecular mechanism of the effect of WRKY on alkali stress of *C. humili* is relatively limited. WRKYs can activate or inhibit the transcription of target genes by binding to the W-box sequence [(T)(T) TGAC (C/T)] of the target gene promoter region. The integration of signaling pathways such as ABA and ROS induces the expression of stress-related genes, thereby regulating the stress response (Goyal et al., 2022). For example, *WRKY33* and *WRKY12* can interact to promote their binding to the downstream target gene RAP2.2 promoter region W-box, activate target gene transcription, and improve the tolerance of *A. thaliana* to hypoxia stress induced by waterflooding (Tang et al., 2020).

On the other hand, plants generate and build up ROS when exposed to non-living environmental stressors. If the ROS levels rise beyond the plant’s capacity to eliminate them, it leads to secondary stress induced by ROS. Maintaining a balance between the generation and removal of ROS, along with the proper regulation of ROS, is crucial for plants to adjust to non-living stress factors. SOD, a key enzyme for scavenging reactive oxygen, is crucial in plants’ antioxidant defense, capable of converting superoxide anion radicals into H_2_O_2_ and O_2_ to eliminate reactive oxygen species (X. Chen et al., 2024; Rehman et al., 2022). SOD expression is stable under normal growth conditions, but changes in SOD expression and activity will occur when plants are under adverse conditions such as high temperature, drought and salt stress (Aleem et al., 2022). Studies have shown that *BpWRKY32* can improve the activities of SOD and POD by inducing the expression of SODs and PODs. The enhanced SOD and POD activities cleared the excess ROS caused by salt stress and improved the salt tolerance (Liu et al., 2024). In this study, based on *MdWRKY115*, which has been reported in *M. pumila* to play an important regulatory role in stress, and combined with transcriptomic data, *ChWRKY29* and *ChWRKY34*, which was identified to respond to alkali stress of *C. humili*, were screened out and verified by yeast single-hybrid experiment. *ChWRKY29* and *ChWRKY34* could directly regulate downstream *ChMSD2* and *ChCSD2* gene expression. Combined with heat map and qPCR analysis, the expression of *ChMSD2* and *ChCSD2* may be up-regulated by *ChWRKY29* and *ChWRKY34* under alkaline treatment. It further affected the antioxidant capacity of plants in response to stress.

According to the transcriptome analysis results, it is worth noting that in the categories of cell components and molecular functions, there are REDOX enzymes related to GO Term. Furthermore, the outcomes of GO and KEGG enrichment analyses revealed a concentration of differential genes in photosynthesis, a plant process utilizing light energy to transform carbon dioxide and water into oxygen and organic substances. In this phase, plants generate substantial quantities of ROS, like oxygen free radicals, which, if not controlled, can lead to severe harm to plant cells. Enzymes with antioxidant properties, like superoxide dismutase (SOD), peroxidase (POD) and ascorbate peroxidase (APX) stand as the primary antioxidant enzymes in plants, collaboratively mitigating oxidative stress. These results suggested that oxidoreductase might play an important role in the resistance of *C. humili* to alkali stress. Metabolic pathways such as carbon metabolism, starch metabolism and sucrose metabolism are related to the synthesis of osmoregulatory substances, suggesting that the synthesis of osmoregulatory substances plays an important role in the alkaline stress of *C. humili*. Free radicals such as ROS are produced in large quantities in plants under stress. Due to peroxidation of the plant cell membrane, membrane permeability increases. On the one hand, osmotic regulation can improve the water absorption capacity of plants by regulating osmotic pressure; on the other hand, osmotic protection can maintain the integrity of cell structure and function, and reduce the damage of stress on plant cells (Hu et al., 2018; Lambers et al., 2006). In conditions of saline-alkali stress, amino acids play a crucial role as precursors for producing in creating secondary metabolites, and their buildup can also improve the elimination of ROS (Fan et al., 2021).

In metabolome analysis, the results of metabolic pathway enrichment were basically consistent with those of transcriptome. The synthesis and activity of antioxidant enzymes are regulated by plant metabolic pathways, and their activity is also related to the concentration of metabolites in plants. Active oxygen species such as hydrogen peroxide can affect the activity of SOD. Antioxidant enzymes, on the other hand, directly affect plant metabolism by coordinating the clearance of reactive oxygen species and the antioxidant defense system of plants. In various metabolic processes, such as sugar metabolism and fat metabolism, superoxide anions and other free radicals are produced, and antioxidant enzymes are essential to maintain the normal progress of metabolism. Both transcriptome and metabolome data indicate the importance of antioxidant enzymes in combating alkali stress in *C. humili*. This study focused on the identification and analysis of the antioxidant enzyme SOD gene family.

Acting as the primary shield for the antioxidant system, the *SOD* gene reacts to diverse environmental cues and shields plants from harmful ROS (Yu et al., 2022). To the best of our knowledge, a comprehensive characterization of the *SOD* gene family in *C. humili* has not been reported. Therefore, the identification of *SOD* gene family members in *C. humili* can provide valuable reference for future functional genetics research, and provide support for genetically improving the stress resistance of *C. humili*. In this study, a total of 9 *ChSODs* were identified from *C. humili*, similar in quantity to *A. thaliana* (8), *Pyrus bretschneideri* (11), *V. vinifera* (10) (Hu et al., 2019), *Hevea brasiliensis* (9) (Yu et al., 2022), *Paeonia lactiflora* (10) (Rafique et al., 2023). There was considerable variation in genome size and the amount of *SOD* genes in these plants, but no substantial dependence on genome size (Rehman et al., 2022). Variations in the number of *SOD* genes across plants could be linked to the process of gene replication. Every recognized *ChCSD* possesses a preserved SOD-Cu domain (Pfam: 00080), while both *ChMSD* and *ChFSD* have a SOD-Fe/Mn domain (Pfam: 02777 and Pfam: 00081). The findings bolster the theory that eukaryotes maintain a high level of conservation in SOD proteins. Numerous works have indicated that *SOD* genes across various species can be classified into three distinct subfamilies. (Hu et al., 2019; Su et al., 2021; Zameer et al., 2022). In this paper, we analyzed the evolutionary relationship between ChSOD protein and SOD protein in five other species, and found that *ChSOD* and *PbSOD* are most closely related, possibly because *C. humili* and *P. bretschneideri* belong to the same family of rosiaceae, and the differences between clades may be related to the different function and diversity of exons/introns and conserved domain. At the same time, there are 1 and 3 motifs in Cu/Zn-SODs and 4 and 9 motifs in Fe/Mn-SODs, indicating that different subfamilies have retained their respective structures during evolution. *ChSOD* gene structure is different even in the same subfamily, which is closely related to the diversity of *ChSOD* gene function.

The duplication of genes plays a crucial role in creating genetic uniqueness across all plants and aids in the adaptation of organisms to environmental shifts (Kondrashov, 2012; Manzoor et al., 2020). Variations in the number of *SOD* genes across various plant species can be attributed to gene replication, and tandem replication and fragment replication play an important role in the diversity, expansion and replication of *SOD* genes (Filiz and Tombuloğlu, 2015; Wang et al., 2016; Zhang et al., 2016). *ChSOD* genes did not replicate in a tandem manner. The hypothesis posits that the repetition of fragments could be the primary catalyst for the development of the *SOD* gene family in *C. humili*. Examining cis-acting components in promoters yields crucial insights for the analysis of *SOD* expression regulation. Examining cis-elements in the *ChSOD* gene promoter uncovered three primary cis-elements associated with light, abiotic stress, and response of hormones. Research indicates the *SOD* gene’s role in various plants’ reactions to non-living stress factors, such as *Dendrobium nobile*, *Zea mays* and *A. thaliana* (Divya et al., 2019; Huang et al., 2020; Liu et al., 2021). We also found more cis-elements related to abiotic stress response in the *ChSOD* gene promoter, which may regulate gene expression under different stresses.

MDA and membrane stability index can reflect the degree of damage to membrane caused by abiotic stress. It was found that alkali stress significantly increased the membrane oxidation of *C. humili* seedlings. SOD, POD and CAT, as enzymatic protection systems in plants, will produce different biochemical reactions when plants are in adversity to alleviate the damage caused by the stressed environment to plants. In this study, MDA content was almost unchanged on the 4^th^ day of treatment and increased significantly on the 8^th^ day, while the activities of three antioxidant enzymes decreased before the 4^th^ day of treatment and then increased on the 8^th^ day. From the perspective of stress resistance index, it was believed that moderate alkali stress was beneficial to the growth of *C. humili* seedlings, while a certain degree of stress would cause damage to the plants. The above three enzymes can play a synergistic role in removing reactive oxygen species and other peroxides when plants grow under adverse conditions, and alleviate the damage caused by environmental stress on plants. In *C. humili*, this synergy is strong (Pearson coefficient > 0.8).

Numerous works have demonstrated the *SOD* gene’s ability to react to a range of non-living stressors. As an illustration, in rapeseed plants, heightened expression of the *MnSOD* gene improves aluminum stress resistance through the amplification of SOD enzyme function. Within *Triticum aestivum* and *A. thaliana*, *TaSOD2* boosts salt tolerance through the regulation of REDOX balance by stimulating NADPH oxidase function. Similarly, overexpression of *TaMnSOD* in *TaMnSOD* decreased MDA content and increased SOD activity in transgenic *Populus przewalskii* under salt stress (Zhou et al., 2024). However, the specific response of *ChSOD* to alkali stress remains unclear. Therefore, qRT-PCR analysis and transcriptome heat maps provide important clues to understand the possible role of *ChSOD* under alkali stress. In this study, the expression levels of 9 *ChSOD* genes also changed significantly under different stress, indicating that these genes play an important regulatory role in stress response, and there may be a certain functional relationship. Some *ChSOD* genes showed different expression patterns, indicating that *ChSOD* played different regulatory roles under alkali stress.

Protein interaction analysis provides insights into the potential regulatory functions and biological roles of SOD proteins. Using a STRING database with a confidence of 0.4 and *A. thaliana* as a template, nine ChSOD proteins were predicted to interact with other family members. Most ChSOD proteins are involved in strong interaction networks, and they may play a regulatory role by forming protein complexes. However, ChMSD2 did not show interaction with other ChSOD proteins, suggesting that this gene may be involved in different metabolic pathways. In addition, CSD has a strong interaction with copper chaperone forsuperoxide dismutase (CCS). CCS is responsible for transferring Cu2+ into the cytoplasm, which increases the concentration of Cu2+ in the cytoplasm and thus promotes the expression of CSD.

## 5 Conclusion

We conducted transcriptomic and metabolomic analysis of *C. humili* under alkali stress. The results showed that *C. humili* has a wide range of metabolic activities under alkali stress, and antioxidant enzymes play an important role in responding to alkali stress. At the same time, we systematically conducted a whole-genome bioinformatics analysis of *ChSODs* in *C. humili*. A total of 9 *ChSODs* were identified from *C. humili*, which were closely related to *Pyrus bretschneideri*. The members of *ChSOD* in the same subfamily were highly similar in motif composition, but the size and distribution of exons/introns were still different. There are a certain number of promoter elements in the *ChSOD* promoter region that are resistant to abiotic stresses, and there is no series replication between *ChSOD* genes, only one fragment replication, fragment repetition may be the main driving force for the evolution of *SOD* gene family in *C. humili*. The results of interspecific collinearity analysis showed that *C. humili* is most closely related to *Malus pumila*. Heat map and qPCR results showed that *ChSOD* played an important role in plant resistance to alkali stress, and homologous genes showed different response patterns. In addition, *WRKY* genes that respond to alkali stress were screened out according to establishment of evolutionary tree with *MdWRKY115* and transcriptome data, and their direct regulatory effects on downstream *ChMSD2* and *ChCSD2* genes were verified by yeast single hybridization experiment. Combined with heat map analysis, qPCR analysis and antioxidant enzyme activity determination, *ChWRKY29* and *ChWRKY34* may be regulated under alkali treatment, and *ChMSD2* and *ChCSD2* expressions are up-regulated, which further affects the antioxidant capacity of plants in response to alkali stress.

## Data Availability

The data presented in the study are deposited in the NCBI repository, BioProject number: PRJNA1260222 (https://www.ncbi.nlm.nih.gov/sra/PRJNA1260222).
