# Supplementary figures and images for: Study on physiological changes and response mechanism of Cerasus humilis under alkali stress
Source: Front Plant Sci. 2025 May 21;16:1586093. doi: 10.3389/fpls.2025.1586093 (PMC12133510; doi:10.3389/fpls.2025.1586093)

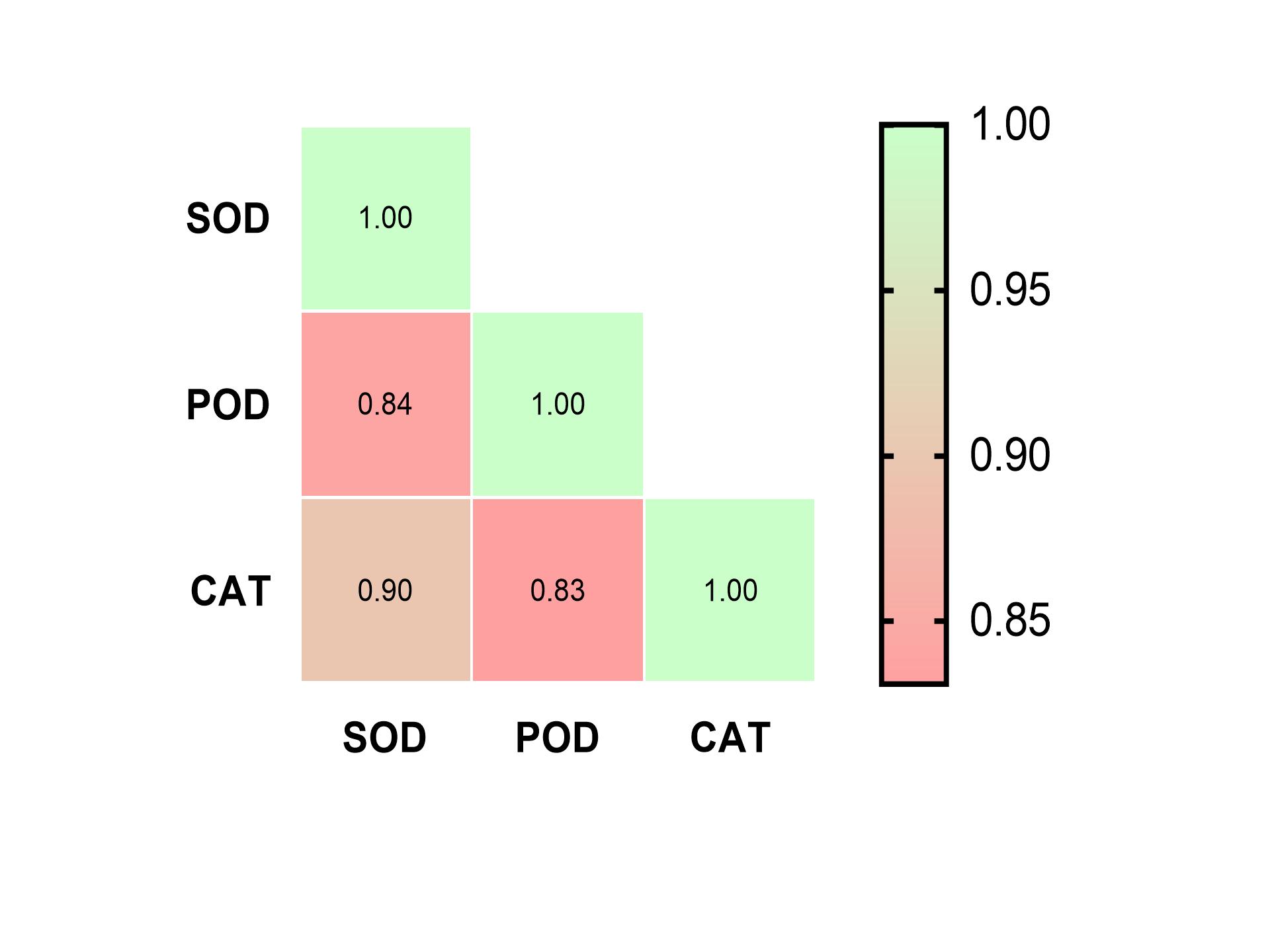

Supplement: Supplementary file 1 [file Image1.jpeg]
